# Incomplete antenatal care despite high coverage: geographic and sociocultural barriers in Lao PDR

**DOI:** 10.3389/fpubh.2025.1625379

**Published:** 2025-07-30

**Authors:** Hyunkyong Kim, Hyejung Han, Khammany Phommachan, Yong Sook Yang, Sung Hye Kim

**Affiliations:** ^1^Yonsei Graduate School of International Studies, Seoul, Republic of Korea; ^2^Center for Global Health Practice, Institute of Human and Society, Hanyang University, Seoul, Republic of Korea; ^3^Department of Public Health Sciences, Graduate School of Hanyang University, Seoul, Republic of Korea; ^4^National Center for Maternal and Child Health (NCMCH), Ministry of Health, Vientiane, Lao People's Democratic Republic; ^5^Development Alliance for Resilience and Inclusion, Vientiane, Lao People's Democratic Republic

**Keywords:** maternal mortality, antenatal care, barriers, equity, Lao PDR

## Abstract

**Background:**

Despite progress in maternal health in Lao PDR, timely access and the completeness of antenatal care (ANC) services remain uneven, particularly in remote provinces with high maternal mortality. Understanding the patterns of ANC utilization and barriers to receiving adequate care is essential to inform targeted interventions.

**Objective:**

This study examined factors associated with early ANC initiation (≤12 weeks’ gestation), adequate ANC (≥4 visits), and the coverage of essential ANC components among women in two northern provinces.

**Methods:**

In February 2023, we conducted a cross-sectional survey in Xiengkhuang and Huaphanh provinces, using stratified three-stage cluster sampling to recruit 380 women with children under 2 years. Face-to-face interviews collected data on ANC timing and frequency, receipt of 13 Ministry-of-Health-defined ANC services, and education on five key pregnancy danger signs. Descriptive statistics summarized utilization, and multivariable logistic regression identified predictors of timely and adequate ANC.

**Results:**

While 95% of participants reported at least one ANC visit and 77.1% had four or more, only 34.5% began ANC within the first trimester. Lack of road access was associated with lower odds of timely and adequate ANC up to 59% (adjusted odds ratio [aOR] for adequate ANC: 0.41; 95% CI: 0.19–0.90). Ethnic minorities, specifically Hmong-Lu Mien women, were associated with 87% reduced odds of having adequate ANC e (aOR: 0.13; 95% CI: 0.02–0.71). Service completeness remained low: just 10.7% reported receiving all essential 13 ANC components, and comprehensive education on all five key pregnancy danger signs was rare (1.5%), and 1.5% reported receiving full education on danger signs; counseling on life-threatening symptoms reached 5.7%. Laboratory services (anemia screening 82.2%, HIV testing 78.4%) and iron supplementation (98%) were common, but only 24.4% reported folic acid provision.

**Conclusion:**

Despite significant improvements in overall ANC coverage in Lao PDR, critical gaps persist in timely initiation and completeness of ANC services, particularly among geographically and ethnically marginalized groups. These findings underscore the urgent need for targeted interventions that address both geographic and cultural barriers and enhance the timeliness and completeness of ANC services to reduce disparities and maximize maternal health benefits in resource-limited settings.

## 1 Introduction

Improving maternal health and reducing maternal mortality have been long-standing global priorities (1, 2), through the Millennium Development Goals (MDGs) (3), and further expanded under the Sustainable Development Goals (SDGs) (4). While significant progress has been made in decreasing global maternal death (5), substantial inequalities persist in healthcare access and outcomes, both between countries and within populations, across different regions, socioeconomic strata, and ethnic groups (6–8). These disparities are often exacerbated in setting with weak health systems (9) characterized by uneven service delivery, resource limitations, and challenges in ensuring comprehensive care (10).

The Lao People’s Democratic Republic (Lao PDR) has also demonstrated substantial progress in maternal health (11), with the maternal mortality ratio declining dramatically from 579 per 100,000 live births in 2000 to 112 in 2023 (12). Furthermore, antenatal care (ANC) coverage reported by the Ministry of Health has significantly increased from 47.6% in 2015 to 107.7% in 2023, largely due to expanded health facilities, skilled personnel, and improved financial protection (13, 14). Despite these achievements, substantial disparities in maternal healthcare persist across different regions and population groups within Lao PDR (15).

To further understand these persistent disparities, particularly concerning ANC utilization and completeness, this study focused on Xiengkhuang and Huaphanh provinces, which represent key geographic and demographic contexts in Lao PDR (16). This study aimed to identify factors associated with timely ANC initiation and adequate ANC, and to assess the coverage and completeness of essential ANC components received by women in these two provinces of Lao PDR.

## 2 Materials and methods

### 2.1 Study setting

This cross-sectional study utilized data collected from a baseline survey conducted from February 12 to 27, 2023 in Xiengkhuang and Huaphanh provinces. This survey was conducted as part of Phase-III (2022–2027) of the “Improving the Quality of Care for Reproductive, Maternal, Newborn, Child and Adolescent Health Project through Strengthening Healthcare System in Lao PDR” project. It was led by the Maternal and Child Health Center of the Lao Ministry of Health (MOH) and Provincial Health Departments, supported by the Korean Foundation for International Healthcare (KOFIH) and contributed to the evaluation of the national maternal and child health improvement program.

Xiengkhuang and Huaphanh provinces are located in the central and northern regions of Lao PDR, covered by mountainous terrain, rolling hills, and grasslands. Their populations are approximately 274,000 and 317,000 people, respectively (17). The study strategically selected districts where comprehensive maternal health services, including cesarean sections, were performed. This criterion was essential to ensure the assessment of antenatal care (ANC) utilization in areas offering a broader spectrum of higher-level healthcare facility availability and specialized obstetric care, thereby enabling the study to investigate ANC patterns within contexts where advanced services are, in principle, accessible.

### 2.2 Sampling design

**Figure 1:**
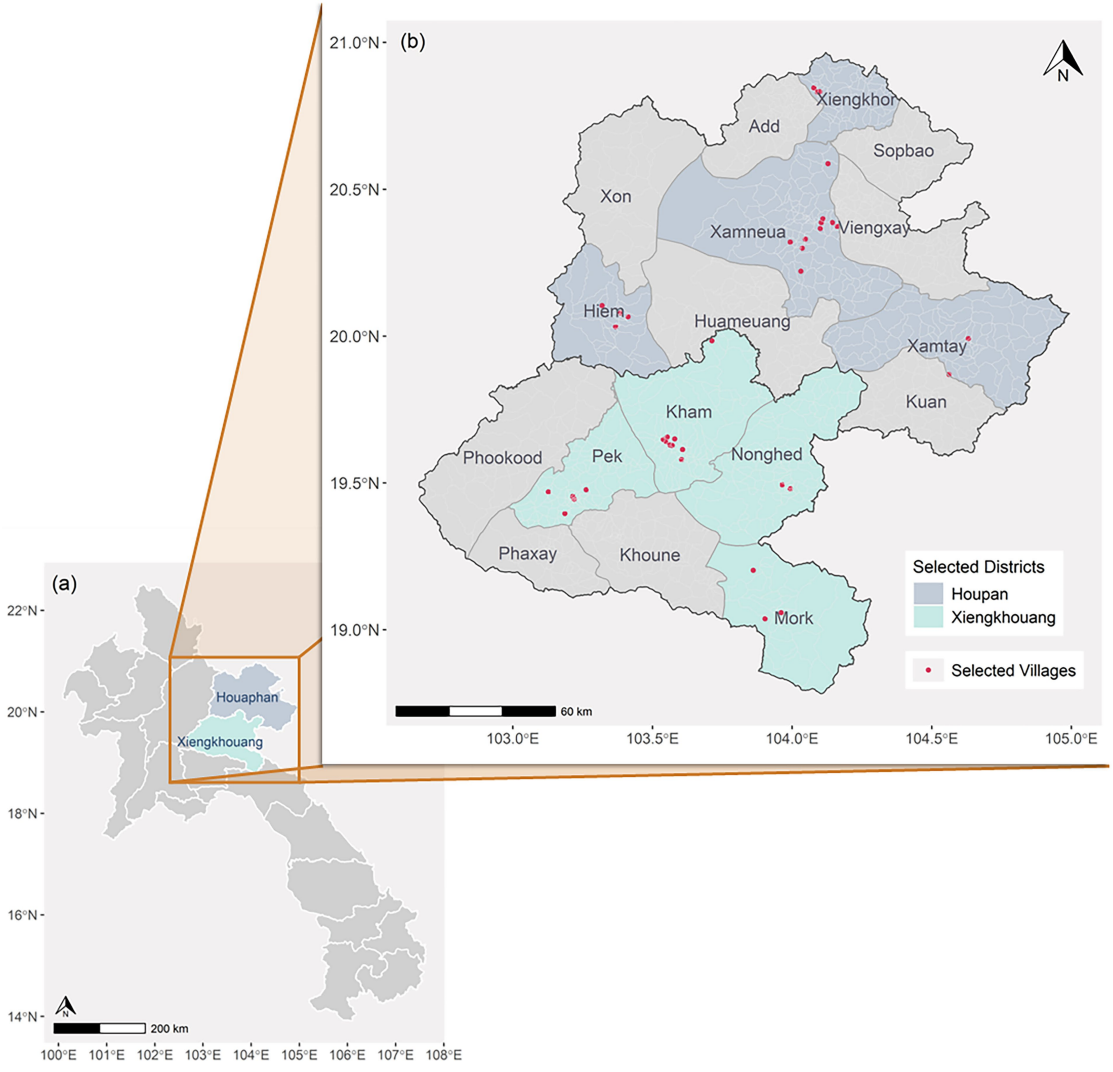
Geographic distribution of selected districts and villages in the surveyed provinces.

The survey employed a three-stage stratified cluster sampling design across two geographic strata (Figure 1). In each province, four districts (where comprehensive maternal health services, including cesarean section services, were available) were included, from which 19 villages were randomly selected as primary clusters (18). Ten households were randomly selected from each village. From each household, one eligible woman was randomly selected, until the total sample of 380 respondents were enrolled. An eligible woman was defined as a female of childbearing age with at least one child aged 0–23 months, ensuring recent experience with maternal health services.

For the sample size computation, a design effect of 2 was applied to account for clustering (19). The calculation was primarily based on achieving statistical power for adequate ANC utilization, assuming an adequate ANC coverage of 65% for ≥4 visits. The estimate was derived from the Provincial Health Department. The sample size was computed using G*Power software (20). With a Z-score of 2.75, the study aimed for 97% statistical power to detect meaningful differences between two provinces in adequate ANC utilization. While this calculation focused on adequate ANC coverage, a more robust approach would have considered other key outcomes, timely ANC initiation and associated factors, incorporating specific assumptions regarding confidence intervals, margin of error, and anticipated non-response rates to ensure comprehensive validity.

### 2.3 Study population

The geographic scope for participant recruitment encompassed selected villages across the two provinces, and we recruited participants who were residing in these communities during the survey implementation.

### 2.4 Data collection

Data were collected through face-to-face-interviewers conducted by eight trained health officers, comprising two officers per team. These officers, from the Provincial Health Department and District Health Office, collaborated with village leaders to announce the survey schedule and ensure community awareness. To ensure the quality of the collected data and the clarity of the questionnaire, a semi-structured questionnaire was initially developed based on a generic household toolkit (21), and its consistency was ensured through a process of translation and back-translation between English and Laos. The questionnaire underwent validation through preliminary testing in a pilot village, which involved approximately 10 female participants according to the availability of participants. This validation process was overseen by supervisors from the National University of Health Science (UHS), who refined the questionnaire’s clarity and relevance for the target population. To further ensure data quality, supervisors from UHS reviewed all questionnaires from each team. During the face-to-face interviews, maternal health books originally provided by the health center were utilized as reference documents to confirm the type of services and the date of service received. For women without health books, they verbally provided related information to interviewers.

The questionnaire was designed to gather comprehensive information on key aspects of maternal health, including socioeconomic characteristics, ANC timing and frequency, the specific services received during ANC sessions, and participants’ awareness of pregnancy danger signs (21).

### 2.5 Data management and analysis

Data entry was executed using Excel with a pre-formatted spreadsheet that featured built-in data validations, dropdown menus, to reduce errors. Once the data was entered, pivot tables were utilized to clean the dataset—identifying duplicates, harmonizing formats, and correcting inconsistencies for a smooth transition to analysis. The data was then processed into a refined format suitable for statistical evaluation, with numerical and categorical responses standardized for consistency. Finally, categorization was achieved using custom scripts, including VBA macros, which systematically grouped responses based on predetermined criteria, ensuring that every entry was classified uniformly and accurately. Missing data were not imputed as it is less than 5% (22).

The collected data were first analyzed using descriptive statistics to summarize the demographic characteristics of participants and their antenatal care (ANC) utilization patterns. These descriptive measures included mostly frequencies and percentages, and means with standard deviations for continuous variables, i.e., participant age. The complex survey design was systematically incorporated into the estimation procedures for all reported proportions and percentages using Stata’s survey (svy) commands with appropriate sampling weights. This methodological approach ensures that the reported estimates accurately represent the target population parameters while maintaining proper standard errors that account for design effects arising from clustering and stratification (23). Chi-square tests were used to compare proportions between ethnic groups in key maternal health indicators (24). Normality testing was not applicable as most of our variables are categorical.

To quantify the associations between various potential determinants and maternal ANC utilization patterns, univariable and multivariable logistic regression models were applied. The study focused two primary outcomes: adequate ANC attendance, defined as four or more ANC visits (≥4 visits), and timely ANC initiation defined as the first ANC visit occurring within the first 12 weeks of pregnancy (≤12 weeks). Odds ratios (ORs) with 95% confidence intervals (CIs) are reported to represent these associations.

To control for potential confounders in the multivariable analysis, a stepwise forward variable selection approach was employed. This approach used thresholds for both inclusion and exclusion of variables set at 0.1 to systematically explore the importance of different variables in the model and to address multicollinearity (25).

The completeness of ANC services was assessed as a key study outcome variable by examining the coverage of specific healthcare services and information provided during ANC sessions, consistent with the study’s objective to assess the coverage of essential ANC components. This assessment included the receipt of 13 essential ANC components outlined by the Ministry of Health, including various laboratory diagnostics (hemoglobin test for anemia screening, blood sugar test, point of care antibody detection tests for syphilis and HIV, and spot urinalysis for protein), nutritional supplementation (iron tablets, calcium carbonate, folic acid), and the provision of at least two tetanus injections. Additionally, comprehensive education on five key pregnancy danger signs was evaluated, specifically covering headaches, high or chronic fever and fatigue, severe abdominal pain, blood or amniotic fluid from vagina, and convulsions.

All statistical analyses were performed using STATA/IC version 15.0 (StataCorp LLC, College Station, TX, USA). While the study employed a three-stage stratified cluster sampling design and applied a design effect of 2 for sample size computation to account for clustering, multilevel analysis was not employed in the regression models. To calculate the intra-cluster correlation coefficient (ICC) for the binary variable indicating whether mothers made four or more antenatal care visits, we began by transforming the aggregated village-level data into individual-level binary responses. Each mother was assigned a value of 1 if she had made four or more visits, and 0 otherwise. We then fitted a mixed-effects logistic regression model with a random intercept for each village to estimate the variance attributable to differences between villages. The ICC was computed using the formula: between-cluster variance divided by the sum of between-cluster variance and the fixed within-cluster variance for logistic models, which is π^2^/3 (26). The resulting ICC was 0.0114, indicating that only about 1.14% of the variation in ANC4 utilization is due to differences between villages.

This low ICC suggests that the clustering effect is minimal, and most of the variation occurs within villages rather than between them. Therefore, while we considered the hierarchical structure of the data, the decision not to adopt a multilevel modeling approach in the main analysis is scientifically justified. The ICC provides reassurance that standard analytical methods are unlikely to be biased by ignoring clustering in this case.

## 3 Results

### 3.1 Participant characteristics

A total of 380 eligible females from 380 households were interviewed, for which demographic data were available (Table 1). The mean age of the participants was 25.9 ± 5.8 years (mean ± standard deviation). Participant ages ranged from 15 to 19 years (12.9%, *n* = 49), 20–29 years (61.8%, *n* = 235), to 30–49 years (25.3%, *n* = 96). Educational attainment varied among participants, with 27 (7.1%) reporting no formal education, 85 (22.4%) having preschool & primary education, 104 (27.4%) completing middle school, and 164 (43.1%) having more than high school education.

**Table 1:** General characteristics of the study participants.

Characteristics	Number (%)
Participant age (years), mean [SD]	25.9 [5.8]
15–19	49 (12.9)
20–29	235 (61.8)
30–49	96 (25.3)
Number of family members	
≤3	16 (4.2)
4–6	198 (52.1)
7–10	130 (34.2)
11≤	36 (9.5)
Education	
No education	27 (7.1)
Preschool & primary	85 (22.4)
Middle school	104 (27.4)
More than high school	164 (43.1)
Having an income-earning job	
Yes	269 (70.8)
No	111 (29.2)
Wealth index (*n* = 371)	
Poorest	76 (20.5)
Second	74 (20.0)
Middle	73 (19.5)
Fourth	74 (20.0)
Highest	74 (20.0)
Religion	
Buddhism	158 (41.6)
Tai Folk	220 (57.9)
Christianity	2 (0.5)
Ethnicity	
Lao	175 (46.1)
Mon-Khmer	65 (17.1)
Hmong-Lu Mien	114 (30.0)
Others	26 (6.8)
Road accessibility	
Yes	191 (50.3)
Partial	83 (21.8)
No	106 (27.9)
Distance to the nearest facility (km)	
<5	90 (23.7)
6–10	130 (34.2)
≥11	160 (42.1)

SD, standard deviation; km, kilometer.

In terms of economic factors, 269 participants (70.8%) reported having an income-earning job. The wealth index, which measures economic status, was distributed across quintiles: Poorest (20.5%, *n* = 76), Second (20.0%, *n* = 74), Middle (19.5%, *n* = 73), Fourth (20.0%, *n* = 74), and Highest (20.0%, *n* = 74) (*n* = 371 due to missing data). The majority of participants (57.9%, *n* = 220) practiced Tai Folk religion, while 41.6% (*n* = 158) practiced Buddhism, and 0.5% (*n* = 2) Christianity. The ethnic background of participants included Lao (46.1%, *n* = 175), Mon-Khmer (17.1%, *n* = 65), Hmong-Lu Mien (30.0%, *n* = 114), and Others (6.8%, *n* = 26). The ethnic minority group collectively comprised 205 participants (53.9%), representing the sum of Mon-Khmer, Hmong-Lu Mien, and Others.

Regarding geographic factors affecting access to care, road accessibility varied: 50.3% (*n* = 191) reported full road access, 21.8% (*n* = 83) had only partial road access, and 27.9% (*n* = 106) lived without road access. The distance to the nearest health facility (km) also posed challenges: 23.7% (*n* = 90) lived <5 km away, 34.2% (*n* = 130) lived 6–10 km away, and 42.1% (*n* = 160) lived ≥11 km away. Most participants (87.9%, *n* = 334) reported receiving education on the danger signs of pregnancy and an equal number (87.9%, *n* = 334) reported institutional childbirth.

### 3.2 Antenatal care (ANC) utilization patterns

Most participants (361, 95%) reported at least one ANC visit during their most recent pregnancy. Specifically, 222 participants (58.4%) reported attending 4–7 ANC visits, and 71 (18.7%) attended more than 8 visits (Table 2). In contrast, 15 participants (3.9%) reported never receiving any ANC, and 4 participants (1.1%) did not know the number of visits they had. Overall, 77.1% of participants had four or more visits. Significant disparities exist in ANC and facility delivery utilization among different ethnic groups. Lao women demonstrate notably higher engagement with maternal health services, with 93.8% attending four or more ANC visits compared to 62.9% among women from other ethnic groups. Additionally, Lao women-initiated ANC earlier with 45.7% doing so versus 24.9% of their counterpart. This pattern extends to childbirth practices, with only 4.6% of Lao women delivering outside health facilities, in contrast to 18.5% among other non-Lao ethnic groups, indicating a substantially higher rate of home birth in those communities.

**Table 2:** ANC and facility delivery service utilization of the study participants.

Variables	Total number (%)	Ethnicity		*p*-value^a^
		Lao number (%)	Other ethnic groups number (%)	
No. of pregnancies				
1	112 (29.5)	58 (33.1)	54 (26.3)	0.225
2	115 (30.2)	54 (30.9)	61 (29.8)	
≥3	153 (40.3)	63 (36.0)	90 (43.9)	
No. of visits to antenatal care services				
None	15 (3.9)	1 (0.6)	14 (6.8)	<0.001
<4	68 (17.9)	9 (5.1)	59 (28.8)	
4–7	222 (58.4)	117 (66.9)	105 (51.2)	
>8 times	71 (18.7)	47 (26.9)	24 (11.7)	
Do not know	4 (1.1)	1 (0.6)	3 (1.5)	
Time of first ANC < 12 weeks				
<3 months	131 (34.5)	80 (45.7)	51 (24.9)	<0.001
>3 months	232 (61.1)	94 (53.7)	138 (67.3)	
Do not know	17 (4.4)	1 (0.6)	16 (7.8)	
Told about the danger signs during pregnancy				
Yes	334 (87.9)	159 (90.9)	175 (85.4)	0.015
No	31 (8.2)	7 (4.0)	24 (11.7)	
Do not know	15 (3.9)	9 (5.1)	6 (2.9)	
Institutional childbirth				
Yes	334 (87.9)	167 (95.4)	167 (81.5)	<0.001
No	46 (12.1)	8 (4.6)	38 (18.5)	

ANC, antenatal care.

a From Chi-square test.

### 3.3 Factors associated with adequate ANC utilization

Univariable analysis revealed several factors associated with attending adequate ANC (≥4 ANC visits) (Table 3). Participants practicing the Tai Folk religion had reduced odds to complete the recommended visits than Buddhists (OR, 0.14; 95% CI, 0.07–0.28). Ethnicity was also significant, with Mon-Khmer and Hmong-Lu Mien participants having reduced odds of adequate ANC (OR: 0.16; 95% CI: 0.07–0.37 and OR: 0.07; 95% CI: 0.04–0.15, respectively).

**Table 3:** Odds ratios of receiving antenatal care at least four times during the last pregnancy in relation to other factors.

Variables	Crude OR (95% CI)	*p*-value	Adjusted OR^b^ (95% CI)	*p*-value
Participant age (years)				
15–19	1.00		1.00	
20–29	1.51 (0.78–2.95)	0.223	0.49 (0.21–1.14)	0.098
30–49	2.84 (1.25–6.47)	0.013	0.61 (0.21–1.76)	0.362
Number of family members				
≤3	1.00		–^c^	
4–6	1.61 (0.49–5.29)	0.434		
7–10	1.06 (0.32–5.54)	0.919		
≥11	0.33 (0.09–1.23)	0.099		
Education				
None	1.00		1.00	
Preschool & primary	3.86 (1.56–9.56)	0.004	1.06 (0.36–3.10)	0.921
Middle school	5.40 (2.18–13.23)	<0.001	1.12 (0.38–3.32)	0.833
More than high school	12.97 (5.19–32.40)	<0.001	1.57 (0.50–4.94)	0.441
Having an income-earning job^a^				
Yes	1.00		–^c^	
No	0.83 (0.50–1.40)	0.488		
Wealth index				
Poorest	1.00		1.00	
Second	1.94 (0.98–3.82)	0.056	1.98 (0.89–4.41)	0.094
Middle	2.97 (1.43–6.14)	0.003	1.88 (0.76–4.64)	0.173
Fourth	6.33 (2.67–15.00)	<0.001	2.58 (0.90–7.39)	0.077
Highest	8.70 (3.36–22.49)	<0.001	1.74 (0.51–5.88)	0.376
Religion				
Buddhism	1.00		1.00	
Tai Folk & others	0.14 (0.07–0.28)	<0.001	1.44 (0.27–7.71)	0.672
Ethnicity				
Lao	1.00		1.00	
Mon-Khmer	0.16 (0.07–0.37)	<0.001	0.21 (0.04–1.16)	0.074
Hmong-Lu mien	0.07 (0.04–0.15)	<0.001	0.13 (0.02–0.71)	0.018
Others	0.51 (0.13–1.98)	0.334	0.47 (0.05–4.00)	0.487
Road accessibility				
Yes	1.00		1.00	
Partial	2.19 (0.92–5.18)	0.076	1.81 (0.63–5.14)	0.268
No	0.24 (0.14–0.42)	<0.001	0.41 (0.19–0.90)	0.025
Distance to the nearest facility (km)				
≤5	1.00		1.00	
6–10	1.19 (0.58–2.44)	0.637	1.67 (0.67–4.14)	0.269
≥11	0.46 (0.25–0.87)	0.017	1.20 (0.47–3.05)	0.700
No. of pregnancies				
1	1.00		–^c^	
2	1.22 (0.63–2.35)	0.550		
≥3	0.72 (0.41–1.28)	0.264		
Timely first ANC				
Yes	1.00		1.00	
No	0.16 (0.08–0.33)	<0.001	0.29 (0.13–0.66)	0.003

km, kilometer.

a *N* = 371.

b Adjusted for all other variables in the table except for the number of family members, having an income-earning job, and the number of pregnancies.

c Variables omitted in a stepwise variable selection and not included in the multivariable analysis.

Road accessibility and distance to the nearest facility were statistically significant factors: participants living in villages without road access (OR: 0.24; 95% CI: 0.14–0.42) or ≥11 km from a health facility (OR: 0.46; 95% CI: 0.25–0.87) showed reduced odds to receive adequate ANC than those who had access. Furthermore, older maternal age (30–49 years) (OR: 2.84; 95% CI: 1.25–6.47) and even minimal educational attainment had increased odds with adequate ANC to their counterpart.

In multivariable analysis, after controlling for potential confounders, Hmong-Lu Mien ethnicity (adjusted OR [aOR]: 0.13; 95% CI: 0.02–0.71), lack of road access (aOR: 0.41; 95% CI: 0.19–0.90), and delayed ANC initiation (aOR: 0.29; 95% CI: 0.13–0.66) remained significant predictors for adequate ANC. Factors including educational attainment, religious affiliation, and distance to a health facility lost their significance after adjustment, suggesting they may operate indirectly or be confounded. The sample size for this analysis was *N* = 371 due to exclusion of missing data.

### 3.4 Timely ANC initiation and associated factors

Among the participants, 131 (34.5%) initiated ANC within the first trimester (≤12 weeks), whereas 232 (61.1%) had their first visit after the recommended period (*N* = 363 due to missing data). Univariable analysis identified that participants aged 30–49 years showed higher odds for initiating timely ANC than those aged 15–29 years (OR: 3.32; 95% CI: 1.51–7.33) (Table 4). Higher educational attainment, particularly education beyond high school (OR: 7.20; 95% CI: 1.62–32.06), and better economic status, especially the Fourth (OR: 2.24; 95% CI: 1.08–4.66) and Highest wealth index (OR: 3.96; 95% CI: 1.91–8.18), were also strongly associated with increased odds for timely ANC initiation. Conversely, participants practicing Tai Folk religion (OR: 0.43; 95% CI: 0.28–0.66) and those of Hmong-Lu Mien ethnicity (OR: 0.20; 95% CI: 0.11–0.38) had reduced odds for timely initiation of ANC. Geographic barrier, lack of road access, (OR: 0.35; 95% CI: 0.20–0.64) and living ≥11 km from the nearest health facility (OR: 0.42; 95% CI: 0.22–0.82) showed reduced odds for timely ANC initiation.

**Table 4:** Odds ratios of having timely first ANC (<12 weeks) during the last pregnancy in relation to other factors.

Variables	Crude OR (95% CI)	*p*-value	Adjusted OR (95% CI)^b^	*p*-value
Participant age (years)				
15–19	1.00		1.00	
20–29	1.53 (0.73–3.18)	0.257	0.90 (0.37–2.16)	0.813
30–49	3.32 (1.51–7.33)	0.003	0.52 (0.59–3.92)	0.389
Number of family members				
≤3	1.00		–^c^	
4–6	0.89 (0.30–2.67)	0.839		
7–10	0.67 (0.22–2.07)	0.492		
≥11	0.31 (0.78–1.22)	0.094		
Education				
None	1.00		1.00	
Preschool & primary	4.25 (0.92–19.72)	0.064	1.58 (0.30–8.20)	0.589
Middle school	4.04 (0.88–18.51)	0.072	1.11 (0.21–5.89)	0.900
More than high school	7.20 (1.62–32.06)	0.010	1.68 (0.32–8.89)	0.539
Having an income-earning job				
Yes	1.00		–^c^	
No	0.62 (0.38–1.02)	0.058		
Wealth index^a^				
Poorest	1.00		1.00	
Second	1.54 (0.73–3.37)	0.257	1.54 (0.67–3.53)	0.311
Middle	1.99 (0.95–4.15)	0.068	1.40 (0.00–3.28)	0.434
Fourth	2.24 (1.08–4.66)	0.031	1.66 (0.70–3.96)	0.252
Highest	3.96 (1.91–8.18)	<0.001	2.20 (0.89–5.45)	0.089
Religion				
Buddhism	1.00		1.00	
Tai Folk & others	0.43 (0.28–0.66)	<0.001	0.76 (0.29–1.99)	0.583
Ethnicity				
Lao	1.00		1.00	
Mon-Khmer	0.65 (0.35–1.18)	0.154	1.63 (0.55–4.86)	0.379
Hmong-Lu Mien	0.20 (0.11–0.38)	<0.001	0.42 (0.14–1.29)	0.130
Others	1.37 (0.60–3.13)	0.455	3.36 (0.96–11.82)	0.059
Road accessibility				
Yes	1.00		1.00	
Partial	1.21 (0.72–2.05)	0.472	1.05 (0.57–1.94)	0.882
No	0.35 (0.20–0.64)	0.001	0.63 (0.30–1.36)	0.239
Distance to the nearest facility (km)				
<5 km	1.00		1.00	
6–10	1.04 (0.49–2.19)	0.560	0.69 (0.37–1.30)	0.257
≥10	0.42 (0.22–0.82)	0.007	0.46 (0.23–0.94)	0.032
No. of pregnancies				
1	1.00		–^c^	
2	1.26 (0.73–2.18)	0.410		
≥3	1.06 (0.63–1.79)	0.834		

ANC, antenatal care; CI, confidence interval; OR, odds ratio; km, kilometer.

a *N* = 371.

b Adjusted for all other variables in the table except for the number of family members, having an income earning job, and the number of pregnancies.

c Variable omitted in a stepwise variable selection and not included in the multivariable analysis.

After multivariable adjustment (*N* = 355 due to missing data), only distance remained significant, with participants living ≥11 km from the nearest health facility having reduced odds to initiate ANC within the recommended timeframe (aOR: 0.46; 95% CI: 0.23–0.94).

### 3.5 Coverage of essential services during ANC visits

An analysis of ANC visits revealed considerable variability in the coverage of essential interventions, both across for service types and among ethnic groups (Table 5). Laboratory diagnostics showed high uptake: 82.2% participants underwent anemia screening, 80.3% received blood sugar testing, and 79.5 and 78.4% were tested for syphilis and HIV/AIDS, respectively. However, only 63.6% of participants received urinalysis for protein detection. Regarding nutritional supplementation, nearly all participants (98.3%) received iron tablets. However, coverage was lower for other supplements, with 66.9% receiving calcium carbonate and only 24.4% receiving folic acid. Notably, disparities were observed by ethnicity, with women from other ethnic group than Lao receiving lower rates of supplementation.

**Table 5:** Service coverage for the type of service provided during ANC visits.

Type of services provided	Number (%)	Ethnicity		*p*-value^a^
		Lao number (%)	Other ethnic groups number (%)	
HIV counseling				
Yes	270 (71.1)	131 (75.3)	139 (72.8)	0.510
No	61 (16.7)	30 (17.2)	31 (16.2)	
Do not know	34 (9.3)	13 (7.5)	21 (11.0)	
Syphilis counseling				
Yes	262 (71.8)	129 (74.1)	133 (69.6)	0.257
No	61 (16.7)	30 (17.2)	31 (16.2)	
Do not know	42 (11.5)	15 (8.6)	27 (14.1)	
Anemia counseling				
Yes	284 (77.8)	143 (82.2)	141 (73.8)	0.105
No	47 (12.9)	20 (11.5)	27 (14.1)	
Do not know	34 (9.3)	11 (6.3)	23 (12.0)	
Blood sugar counseling				
Yes	269 (73.7)	133 (76.4)	136 (71.2)	0.191
No	60 (16.4)	29 (16.7)	31 (16.2)	
Do not know	36 (9.9)	12 (6.9)	24 (12.6)	
Urine protein counseling				
Yes	237 (64.9)	118 (67.8)	119 (62.3)	0.345
No	88 (24.1)	41 (23.6)	47 (24.6)	
Do not know	40 (11.0)	15 (8.6)	25 (13.1)	
Iron tablets provided				
Yes	359 (98.4)	173 (99.4)	186 (97.4)	0.289
No	5 (1.4)	1 (0.6)	4 (2.1)	
Do not know	1 (0.3)	0 (0.0)	1 (0.5)	
Folic acid provided				
Yes	89 (24.4)	63 (36.2)	26 (13.6)	<0.001
No	255 (69.9)	101 (58.1)	154 (80.6)	
Do not know	21 (5.7)	10 (5.7)	11 (5.8)	
Calcium carbonate provided				
Yes	244 (66.9)	144 (82.8)	100 (52.4)	<0.001
No	115 (31.5)	29 (16.7)	86 (45.0)	
Do not know	6 (1.6)	1 (0.5)	5 (2.6)	
Deworming tablets provided				
Yes	270 (74.0)	146 (83.9)	124 (64.9)	<0.001
No	89 (24.4)	27 (15.5)	62 (32.5)	
Do not know	6 (1.6)	1 (0.6)	5 (2.6)	
Tetanus injection of mothers				
No injection	32 (8.8)	3 (1.7)	29 (15.2)	<0.001
Less than 2 times	37 (10.1)	8 (4.6)	29 (15.2)	
3–4 times	51 (14.0)	18 (10.3)	33 (17.3)	
5 times	244 (66.9)	145 (83.3)	99 (51.8)	
Do not know	1 (0.3)	0 (0.0)	1 (0.5)	
HIV test				
Yes	286 (78.4)	153 (87.9)	133 (69.6)	<0.001
No	57 (15.6)	14 (8.1)	43 (22.5)	
Do not know	22 (6.0)	7 (4.0)	15 (7.9)	
Syphilis test				
Yes	290 (79.5)	159 (91.4)	131 (68.6)	<0.001
No	44 (12.0)	4 (2.3)	40 (21.0)	
Do not know	31 (8.5)	11 (6.3)	20 (10.4)	
Anemia test				
Yes	300 (82.2)	164 (94.3)	136 (71.2)	<0.001
No	45 (12.3)	3 (1.7)	42 (22.0)	
Do not know	20 (5.5)	7 (4.0)	13 (6.80)	
Blood sugar test				
Yes	293 (80.3)	163 (93.7)	130 (68.1)	<0.001
No	53 (14.5)	7 (4.0)	46 (24.1)	
Do not know	19 (5.2)	4 (2.3)	15 (7.8)	
Urine test				
Yes	232 (63.6)	122 (70.1)	110 (57.6)	0.040
No	99 (27.1)	40 (23.0)	59 (30.9)	
Do not know	34 (9.3)	12 (6.9)	22 (11.5)	
Height measure				
Yes	361 (98.9)	173 (99.4)	188 (98.4)	0.147
No	3 (0.8)	0 (0.0)	3 (1.6)	
Do not know	1 (0.3)	1 (0.6)	0 (0.0)	
Weight measure				
Yes	361 (98.9)	173 (99.4)	188 (98.4)	0.147
No	3 (0.8)	0 (0.0)	3 (1.6)	
Do not know	1 (0.3)	1 (0.6)	0 (0.0)	
Blood pressure				
Yes	360 (98.6)	173 (99.4)	187 (97.9)	0.252
No	3 (0.8)	0 (0.0)	3 (1.6)	
Do not know	2 (0.6)	1 (0.6)	1 (0.5)	
Nutrition counseling				
Yes	342 (93.7)	167 (96.0)	175 (91.6)	0.205
No	15 (4.1)	4 (2.3)	11 (5.8)	
Do not know	8 (2.2)	3 (1.7)	5 (2.6)	
Told about danger signs of pregnancy				
Yes	334 (91.5)	159 (91.4)	175 (91.6)	0.269
No	19 (5.2)	7 (4.0)	12 (6.3)	
Do not know	12 (3.3)	8 (4.6)	4 (2.1)	
Told what to do for danger signs (*N* = 334, %)				
Yes	311 (93.1)	155 (97.4)	156 (89.1)	0.007
No	6 (1.8)	2 (1.3)	4 (2.3)	
Do not know	17 (5.1)	2 (1.3)	15 (8.6)	

ANC, antenatal care.

^a^From Chi-square test.

Despite the widespread provision of individual services, only 10.7% of participants received all 13 components of the MOH’s Essential Health Service Package. The figure was higher among Lao women (20.1%), compared to 2.1% among women from other ethnic groups, indicating substantial gaps and ethnic disparities in the completeness of ANC service delivery.

Regarding counseling services, Table 5 shows varying rates of receipt. For HIV counseling, 71.1% of participants reported receiving it. Similarly, 71.8% reported syphilis counseling, 77.8% reported anemia counseling, 73.7% reported blood sugar counseling, and 64.9% reported urine protein counseling. Nutrition counseling was reported by 93.7% of participants. Furthermore, 91.5% of participants reported being told about danger signs of pregnancy, and of those, 93.1% reported being told what to do for danger signs. These counseling rates also showed ethnic disparities in some areas, including lower rates among non-Lao ethnic groups for folic acid provision (13.6% vs. 36.2% for Lao women), calcium carbonate (52.4% vs. 82.8%), tetanus injections (51.8% vs. 83.3%), HIV testing (69.6% vs. 87.9%), syphilis testing (68.6% vs. 91.4%), anemia testing (71.2% vs. 94.3%), blood sugar testing (68.1% vs. 93.7%), urine testing (57.6% vs. 70.1%), and being told what to do for danger signs (89.1% vs. 97.4%).

### 3.6 Awareness of pregnancy danger signs

Education on the danger signs of pregnancy during ANC was inconsistent (Table 6). Of the 334 participants who reported received counseling, 50.6% reported being informed about headaches, and 71.6% reported being informed about blood or amniotic fluid coming out of the vagina. Counseling on several critical danger signs was markedly insufficient:5.7% of participants reported being informed about convulsions, 37.4% reported being informed about high or chronic fever and fatigue, and 60.5% reported being informed about severe abdominal pain. Only 1.5% of the participants reported receiving comprehensive education on all five key danger signs. Chi-square analysis revealed significant disparities in antenatal danger sign education, with Lao women reporting receiving more information than ethnic minority women. Notable differences were observed in awareness of high or chronic fever (47.2% for Lao vs. 28.6% for others), blood or amnionic fluid coming out of the vagina (83.7% for Lao vs. 60.6% for others), and convulsions (8.8% for Lao vs. 2.9% for others), while no significant differences were found for headache or abdominal pain.

**Table 6:** Details of the danger signs of pregnancy explained during ANC visits for their last pregnancy.

Danger signs of pregnancy explained	Number (%)	Ethnicity		*p*-value^a^
		Lao number (%)	Other ethnic groups number (%)	
Headache				
Yes	169 (50.6)	81 (50.9)	88 (50.3)	0.904
No	165 (49.4)	78 (49.1)	87 (49.7)	
High or chronic fever, very tired				
Yes	125 (37.4)	75 (47.2)	50 (28.6)	<0.001
No	209 (62.6)	84 (52.8)	125 (71.4)	
Severe abdominal pain				
Yes	202 (60.5)	96 (60.4)	106 (60.6)	0.971
No	132 (39.5)	63 (39.6)	69 (39.4)	
Blood or amniotic fluid coming out of the vagina				
Yes	239 (71.6)	133 (83.7)	106 (60.6)	<0.001
No	95 (28.4)	26 (16.4)	69 (39.4)	
Convulsions				
Yes	19 (5.7)	14 (8.8)	5 (2.9)	0.019
No	315 (94.3)	145 (91.2)	170 (97.1)	

ANC, antenatal care.

a From Chi-square test.

## 4 Discussion

This study aimed to identify factors associated with timely antenatal care (ANC) initiation and adequate ANC, and to assess the coverage of essential ANC components received by women in Xiengkhuang and Huaphanh provinces of Lao PDR. Our findings revealed a complex picture of ANC utilization while overall access appears high, with 95% participants reporting at least one ANC visit and 77.1% achieving four or more visits, significant challenges remain in timely initiation, with only 34.5% initiating ANC within the recommended first trimester. We observed pronounced disparities related to geographic and ethnic factors, where a lack of road access significantly reducing the odds of timely and adequate ANC and Hmong-Lu Mien women experiencing 87% reduced odds of adequate ANC care. Furthermore, a critical disconnect emerged between the quantity of visits and the completeness of services: only 10.7% of participants received all 13 essential ANC components outlined by the Ministry of Health (MOH), and comprehensive education on all five key pregnancy danger signs was rare (1.5%). While basic services including laboratory tests and iron supplementation were commonly provided, critical gaps existed in folic acid provision (24.4%) and counseling on life-threatening symptoms, convulsions (5.7%). Although the MOH data, through District Health Information System 2 (DHIS2), indicate a dramatic increase in overall ANC coverage from 47.6% in 2015 to 107.7% in 2023, our findings suggest that this quantitative improvement has not translated into comprehensive care, highlighting a fundamental challenge where services are often incomplete despite apparent access. Social Indicator Surveys report that ANC4 coverage rose from 36.9% in 2012 to 71.6% in 2023 (27, 28), yet timely ANC uptake plateaued at 56% in 2023—and our study found even lower rates in Xiengkhuang and Huaphanh, dominated by ethnic minorities and with poorer road access than the national average—highlighting a persistent, fundamental challenges in the region (29).

The observed patterns of inadequate and delayed ANC, particularly among women from non-Lao ethnic groups, reflect a complex interplay of both structural healthcare deficiencies and embedded socio-cultural dynamics (30). From a structural perspective, our study highlights critical rural service gaps, most notably significant impact of geographic barriers. Lack of road access and increased distance to health facilities consistently reduced the likelihood of both timely ANC initiation and adequate follow-up visits. This findings aligns with existing literature on health care access in challenging terrains: Given that the rugged terrain and dispersed rural communities characteristics in Lao PDR (31), simply expanding the number of facilities may not be sufficient. Instead, efforts must prioritize enhancing the completeness and quality of services for individuals who already face significant challenges in overcoming these access barriers (32, 33). This implies a need for strategies that bring comprehensive, quality care closer to remote populations or improve the transport infrastructure to facilitate access (34).

Beyond structural issues, socio-cultural dynamics significantly contribute to the observed disparities (35, 36). Our results demonstrate that ethnic minorities, particularly, Hmong-Lu Mien women, consistently demonstrated lower ANC utilization, indicating potential limitations in reproductive autonomy and agency within their communities. This is further exacerbated by factors including remote residence, limited means of transportation, lower education attainment, and the exclusion of females from decision-making practices, which collectively limit health literacy and access to care (37, 38). While our study did not directly assess provide behavior, the pronounced ethnic disparities in service uptake and danger sign awareness suggest a potential impact of culturally inappropriate practices by healthcare providers or suboptimal interpersonal skills among medical personnel. This inference is supported by other research indicating that such factors can hinder effective service delivery and patient satisfaction for marginalized groups (39, 40). Therefore, our findings underscore the importance of delivering maternal healthcare that is not only clinically comprehensive but also culturally sensitive and responsive to the specific needs of diverse ethnic groups (41). The challenges identified in our analysis, particularly the gaps in maternal education regarding danger signs and the complete range of essential ANC services, disproportionately affect marginalized populations (42). Previous research in Laos also emphasizes that deficiencies in providers’ attitudes, communication skills, and cultural responsiveness further hinder effective service delivery and patient satisfaction (43). This highlights a critical need to invest in training and support for healthcare providers to ensure they are equipped to offer high-standard, patient-centered care that addresses both the clinical and cultural needs of the population (41).

Our findings revealed notable inconsistencies in the completeness of essential services provided during ANC visits. While certain components including laboratory diagnostics achieved high coverage (78–82% for most tests) and nearly every participant (98.3%) received iron supplementation, other critical services were severely lacking. For instance, only 24.4% of the participants reported receiving folic acid supplementation. While recall bias could partly contribute to this low figure, as folic acid is often provided together with iron, this deficiency, along with incomplete danger sign education, signals the need for improvement to prevent adverse maternal outcomes. This discrepancy unequivocally demonstrates that a quantitative increase in the number of ANC visits does not automatically translate into a comprehensive provision of all necessary services, echoing similar challenges reported in other settings with high ANC coverage but low quality (44).

Most alarmingly, our study identified a profound deficiency in education regarding pregnancy danger signs. A staggering 94.3% of participants reported not receiving information about convulsions, a life-threatening symptom of eclampsia requiring immediate intervention (45). Similarly, significant gaps also existed for other critical warning signs: 62.6% did not receive guidance on high or chronic fever and fatigue, and 39.5% were uninformed about severe abdominal pain. The finding that only 1.5% of participants recalled receiving comprehensive education on all five key danger signs, highlights a critical weakness in the current ANC service provision (46). The substantial lack of comprehensive danger sign education is a severe impediment, as it directly limits women’s ability to recognize complications and seek timely emergency care, thereby undermining broader efforts to reduce maternal and neonatal mortality (47).

Our results suggest that, while initiatives aimed at expanding ANC coverage in Lao PDR, including the Free Maternal Health Services Policy and the Health and Nutrition Services Access Project (HANSA), have successfully increased access (48), these efforts must now be rigorously paired with robust quality assurance measures. Upgrading service quality is paramount, encompassing standardized health education, consistent delivery of all components of the Essential Health Service Package, and improved nutritional supplementation (34, 49). Furthermore, enhanced provider communication and cultural competency are crucial not only for improving the overall patient experience but also ensuring that each ANC visit delivers its maximum clinical benefits (50). In terms of practical implications, healthcare providers need clear protocols and consistent training to ensure that all 13 essential components are delivered during every ANC visit. For policy, this signifies a shift in focus from merely increasing visit numbers to implementing and rigorously monitoring quality standards. This includes strategic investments in comprehensive training programs for healthcare workers, particularly those operating in remote and underserved areas, and alongside with the development of culturally appropriate health education materials.

Future research and policy initiatives should explore innovative approaches to integrating service delivery, including targeted provider training and structured protocols that ensure every ANC contact comprehensively addresses both clinical and educational needs, particularly for women facing significant geographic and sociocultural barriers. By simultaneously advancing access and the completeness and quality of care, the Lao PDR’s maternal healthcare system can more effectively reduce disparities and improve outcomes for its most vulnerable populations. Future research also could employ mixed methods approaches to deeply explore the reasons behind the identified quality gaps, incorporating provider perspectives and examining structural barriers within the health system to inform more precise, targeted interventions.

## 5 Strength and limitations

This study demonstrates several significant strengths. Firstly, its timely data collection in February 2023 provides recent and relevant insights into ANC utilization within the important geographic and demographic contexts of Xiengkhuang and Huaphanh provinces in Lao PDR. Secondly, the research focused on a targeted study population of females with recent maternal health experience (at least one child aged 0–23 months), ensuring the direct relevance of collected data to contemporary maternal health services. Most critically, the study offered a comprehensive assessment of ANC services by examining not just visit frequency, but also the receipt of 13 essential ANC components outlined by the Ministry of Health and comprehensive education on five key pregnancy danger signs. This provided critical insights into service completeness and allowed for the identification of disparities among marginalized groups, offering actionable implications for targeted interventions.

Despite these strengths, this study is subject to several methodological limitations, though specific measures were implemented to mitigate their impact. One limitation is the reliance on self-reported data, which introduces potential recall bias. However, this was addressed by targeting females with recent maternal health experience (children aged 0–23 months) and utilizing maternal health books for double-checking information, alongside the use of a validated questionnaire. The cross-sectional study design limits the establishment of causal relationships; however, the study’s objective was to identify factors associated with ANC patterns and assess coverage, for which the chosen logistic regression models were appropriate.

Another limitation pertains to generalizability. While a robust stratified three-stage cluster sampling design ensured representativeness within the selected Xiengkhuang and Huaphanh provinces, the non-probability-based selection of villages for the entire country may limit broader generalizability of findings to the whole of Lao PDR. Finally, multilevel analysis was not employed in the regression models and the negligible Intra-class Correlation Coefficient (ICC) observed, which suggested that the clustering effect on the outcomes was minimal and did not warrant a more complex hierarchical modeling approach.

## 6 Conclusion

The study primarily aimed to identify factors associated with timely ANC initiation and adequate ANC, and to assess the coverage and the completeness of essential ANC components among women in Xiengkhuang and Huaphanh provinces, Lao PDR. Our findings reveal a nuanced picture: while overall ANC utilization is very high, with nearly all participants attending at least one ANC visit and a substantial majority having four or more visits, critical gaps persist in timely initiation, as only a minority-initiated ANC within the recommended first trimester.

A significant disconnect was found between visit quantity and the completeness of services received. Alarmingly, only a very small proportion of participants reported receiving all 13 essential ANC components outlined by the Ministry of Health, and comprehensive education on all five key pregnancy danger signs was exceptionally rare, reported by almost no participants. This highlights a critical weakness in the current ANC service provision, limiting women’s ability to recognize complications and seek timely emergency care.

Furthermore, the study identified geographic barriers, a lack of road access, which substantially reduced the odds of adequate ANC. Ethnic disparities were also prominent, with Hmong-Lu Mien women experiencing significantly reduced odds of adequate ANC care. These findings underscore the urgent need to shift focus from merely increasing ANC visit numbers to enhancing the completeness and quality of each visit, particularly among geographically and ethnically marginalized groups. Implementing robust quality assurance measures, upgrading service quality through standardized health education, ensuring consistent delivery of all components of the Essential Health Service Package, and improving nutritional supplementation are essential to maximize maternal health benefits and reduce disparities in resource-limited settings.

## Data Availability

The raw data supporting the conclusions of this article will be made available by the authors without undue reservation.
